# Single and combined use of the platelet-lymphocyte ratio, neutrophil-lymphocyte ratio, and systemic immune-inflammation index in gastric cancer diagnosis

**DOI:** 10.3389/fonc.2023.1143154

**Published:** 2023-03-30

**Authors:** Jingliang Zhang, Li Zhang, Shusheng Duan, Zhi Li, Guodong Li, Haiyan Yu

**Affiliations:** ^1^ The First Clinical Medical School, Shanxi Medical University, Taiyuan, China; ^2^ Department of Gastroenterology, Taiyuan Central Hospital of Shanxi Medical University, Taiyuan, China; ^3^ Department of Hematology, Taiyuan Central Hospital of Shanxi Medical University, Taiyuan, China; ^4^ Department of Gastroenterology Surgery, Taiyuan Central Hospital of Shanxi Medical University, Taiyuan, China

**Keywords:** gastric cancer, diagnosis, systemic immune-inflammation index, neutrophil-lymphocyte ratio, platelet-lymphocyte ratio

## Abstract

**Introduction:**

The platelet-lymphocyte ratio (PLR), neutrophil-lymphocyte ratio (NLR), and systemic immune-inflammation index (SII) are markers for systemic inflammatory responses and have been shown by numerous studies to correlate with the prognosis of gastric cancer (GC). However, the diagnostic value of these three markers in GC is unclear, and no research has examined them in combination. In this study, we investigated the value of the PLR, NLR, and SII individually or in combination for GC diagnosis and elucidated the connection of these three markers with GC patients’ clinicopathological features.

**Methods:**

This retrospective study was conducted on 125 patients diagnosed with GC and 125 healthy individuals, whose peripheral blood samples were obtained for analysis. The preoperative PLR, NLR, and SII values were subsequently calculated.

**Results:**

The results suggest that the PLR, NLR, and SII values of the GC group were considerably higher than those of the healthy group (all P ≤ 0.001); moreover, all three parameters were notably higher in early GC patients (stage I/II) than in the healthy population. The diagnostic value of each index for GC was analyzed using receiver operating characteristic (ROC) curve analysis and area under the curve (AUC) calculation. The diagnostic efficacy of the SII alone (AUC: 0.831; 95% confidence interval [CI], 0.777–0.885) was expressively better than those of the NLR (AUC: 0.821; 95% CI: 0.769–0.873, P = 0.017) and PLR (AUC: 0.783; 95% CI: 0.726–0.840; P = 0.020). The AUC value of the combination of the PLR, NLR, and SII (AUC: 0.843; 95% CI: 0.791–0.885) was significantly higher than that of the combination of the SII and NLR (0.837, 95% CI: 0.785–0.880, P≤0.05), PLR (P = 0.020), NLR (P = 0.017), or SII alone (P ≤ 0.001). The optimal cut-off values were determined for the PLR, NLR, and SII using ROC analysis (SII: 438.7; NLR: 2.1; PLR: 139.5). Additionally, the PLR, NLR, and SII values were all meaningfully connected with the tumor size, TNM stage, lymph node metastasis, and serosa invasion (all P ≤ 0.05). Elevated levels of the NLR and SII were linked to distant metastasis (all P ≤ 0.001).

**Discussion:**

These data suggest that the preoperative PLR, NLR, and SII could thus be utilized as diagnostic markers for GC or even early GC. Among these three indicators, the SII had the best diagnostic efficacy for GC, and the combination of the three could further improve diagnostic efficiency.

## 1 Introduction

A report published by the International Agency for Research on Cancer, the 2020 update on the global burden of cancer, showed that gastric cancer (GC) has the fifth highest incidence among all cancers with a mortality rate that ranks fourth in cancer-related deaths worldwide (1).In China, GC is the most common gastrointestinal tumor (2). The 5-year survival rate for early-stage GC after operation surpasses 97% (3). Unfortunately, because the early symptoms of GC are atypical, patients with GC are usually detected in the middle and late stages, with the 5-year survival rate being poor (4, 5). Therefore, diagnosing GC as early as possible is key to effective treatment and prognosis. Currently, endoscopy is the most common and effective method for diagnosing GC (6), but it is not suitable for mass screening of GC because of its invasive nature, low tolerability, and high cost. Moreover, the GC detection rate heavily depends on the endoscopist’s level of practice. Therefore, identifying a noninvasive, inexpensive, easily accessible, specific, and sensitive biological indicator is crucial for the early diagnosis of GC.

The immune inflammatory response can promote blood vessel growth, stimulate tumor cell proliferation, invasion, and metastasis, and decrease immunomodulatory responses (7), which are directly linked to the occurrence and development of tumors (8, 9). The platelet-lymphocyte ratio (PLR), neutrophil-lymphocyte ratio (NLR), and systemic immune-inflammation index (SII) are biological indicators that can reflect the status of systemic immune inflammation and are easy to measure and calculate (10). According to previous studies, the PLR, NLR, and SII are linked to the tumor-node-metastasis (TNM) stage, lymph node metastasis, and the invasion depth of GC. They can potentially be used as indicators to evaluate prognosis in GC patients (10–12). The SII combines three types of inflammatory cells, platelets, neutrophils, and lymphocytes, to more comprehensively exhibit the balance between the inflammatory status and host immune response (13, 14). The SII has a significantly higher predictive power than the PLR and/or NLR for survival in GC patients (10, 15–17). Recent research has shown the value of the PLR and NLR in diagnosing GC (18, 19), but the diagnostic value of the SII is unclear. No studies have reported results of combining the PLR, NLR, and SII.

In this study, we investigated the value of the PLR, NLR, and SII, individually or in combination, in diagnosing GC. This work can offer new insight into the early diagnosis of GC.

## 2 Materials and methods

### 2.1 Participants

We retrospectively examined 125 GC patients who were originally diagnosed at Taiyuan Central Hospital of Shanxi Medical University between May 2017 and March 2022. The 8^th^ edition of the American Joint Committee on Cancer (AJCC)/TNM tumor classification system was used to classify the tumors (20). The enrollment criteria for patients were as follows: (a) diagnosis with the testing of tissue acquired during gastroscopy and verified by postoperative pathology; (b) no radiotherapy, chemotherapy, or immunotherapy performed prior to surgery; and (c) available pretreatment routine blood indicators. The exclusion criteria for candidates were as follows: (a) preoperative combination of serious infectious, autoimmune, or cardiovascular diseases; (b) treatment with antiplatelet medication or statins within 3 months prior to blood examination; (c) received anti-inflammatory and/or blood transfusion therapy within 1 month prior to blood examination; (d) recurrent GC; and (e) combined hematological system diseases and history of other systemic malignancies within 5 years. We enrolled 125 patients in the GC group based on these criteria, which comprised 95 men and 30 women (mean age: 61.91 ± 10.82 years; range: 26–82 years).

For the healthy control (HC) group, we chose 125 healthy subjects who visited the hospital for routine physical examination. They had no history of treatment with antiplatelet therapy, inflammatory disease, autoimmune disease, hematological disease, or cancer. The HC group had 87 men and 38 women (mean age: 59.86 ± 13.32 years; range: 35–89 years).

This study was approved by the Ethics Committee of Taiyuan Central Hospital of Shanxi Medical University (2022010). Because the protocol involved a retrospective study, informed consent was waived, and patient data were treated confidentially.

### 2.2 Methods

All participants had 2 mL of venous blood drawn from the cubital vein on the day of admission or early the next morning with an empty stomach for complete blood cell count measurement. The total platelet, absolute neutrophil, total white blood cell (WBC), and absolute lymphocyte counts were acquired using a blood cell analyzer (MaiRui BC6800 PLUS,Shenzhen, China). The SII was determined by the formula SII = (P × N)/L, where L, N, and P are the lymphocyte, neutrophil, and platelet counts, respectively. The absolute neutrophil and lymphocyte values were used to calculate the NLR values. The total number of platelets and absolute values of lymphocytes were used to calculate the PLR values.

### 2.3 Statistical analysis

Data were analyzed using MedCalc version 20.0 (MedCalc Software, Mariakerke,Belgium) and SPSS 25.0 (SPSS, Chicago, IL, USA) statistical software. Measures obeying normality are expressed as the mean ± standard deviation (SD). Non-normal values are expressed as the median and quartiles. Comparisons between groups were carried out using the Mann-Whitney U-test or t-test. The chi-square test was used to evaluate the categorical variables. The inflammatory indicator levels were compared between groups using the Kruskal-Wallis test. Intergroup comparisons were performed using Bonferroni correction. The diagnostic values of the PLR, NLR, and SII alone and in combination were compared by plotting the receiver operating characteristic (ROC) curve, and the optimal cut-off values were determined for the three inflammatory markers. Statistical significance was defined as *P*-values less than 0.05.

## 3 Results

### 3.1 Laboratory indicators for both groups

Statistical data, such as sex and age, and the levels of various serum biomarkers of the two groups of participants in this study are shown in **Table 1**. There were no statistical differences in sex or age between the GC and control groups. Compared with the control group, GC patients had significantly higher PLR, NLR, and SII values, higher WBC platelet and neutrophil counts, and a lower lymphocyte count, all of which were statistically significant (*P* < 0.05).

**Table 1:** Laboratory values of gastric cancer patients and healthy controls.

Variable	Gastric cancer group (n=125)	Healthy control group (n=125)	*P*-value
n	125	125	
Age (years)	61.91 ± 10.28	59.86 ± 13.32	0.173^a^
Sex (male, %)	95 (76.0%)	87 (69.6%)	0.256^b^
WBCs (10^9^/L)	6.80 ± 1.94	6.32 ± 1.49	0.029^a^
Lymphocytes (10^9^/L)	1.66 ± 0.59	2.14 ± 0.64	< 0.001^a^
Platelets (10^9^/L)	253.02 ± 75.33	217.89 ± 44.60	< 0.001^a^
Neutrophils (10^9^/L)	4.17 ± 1.64	3.22 ± 0.79	< 0.001^a^
SII	585.23 (397.42, 843.50)	339.24 (280.64, 392.33)	< 0.001^c^
NLR	2.26 (1.80, 3.17)	1.52 (1.23, 1.85)	< 0.001^c^
PLR	152.31 (110.00, 208.33)	103.33 (87.43, 125.50)	< 0.001^c^

WBCs, white blood cells; SII, systemic immune-inflammation index; NLR, neutrophil-lymphocyte ratio; PLR, platelet-lymphocyte ratio.

a Difference between groups was tested by two-sample t-test.

b Difference between groups was tested by chi‐square test.

c Difference between groups was tested by Mann-Whitney U‐test.

### 3.2 Diagnostic value of the PLR, NLR, and SII alone and in combination for GC

**Figure 1:**
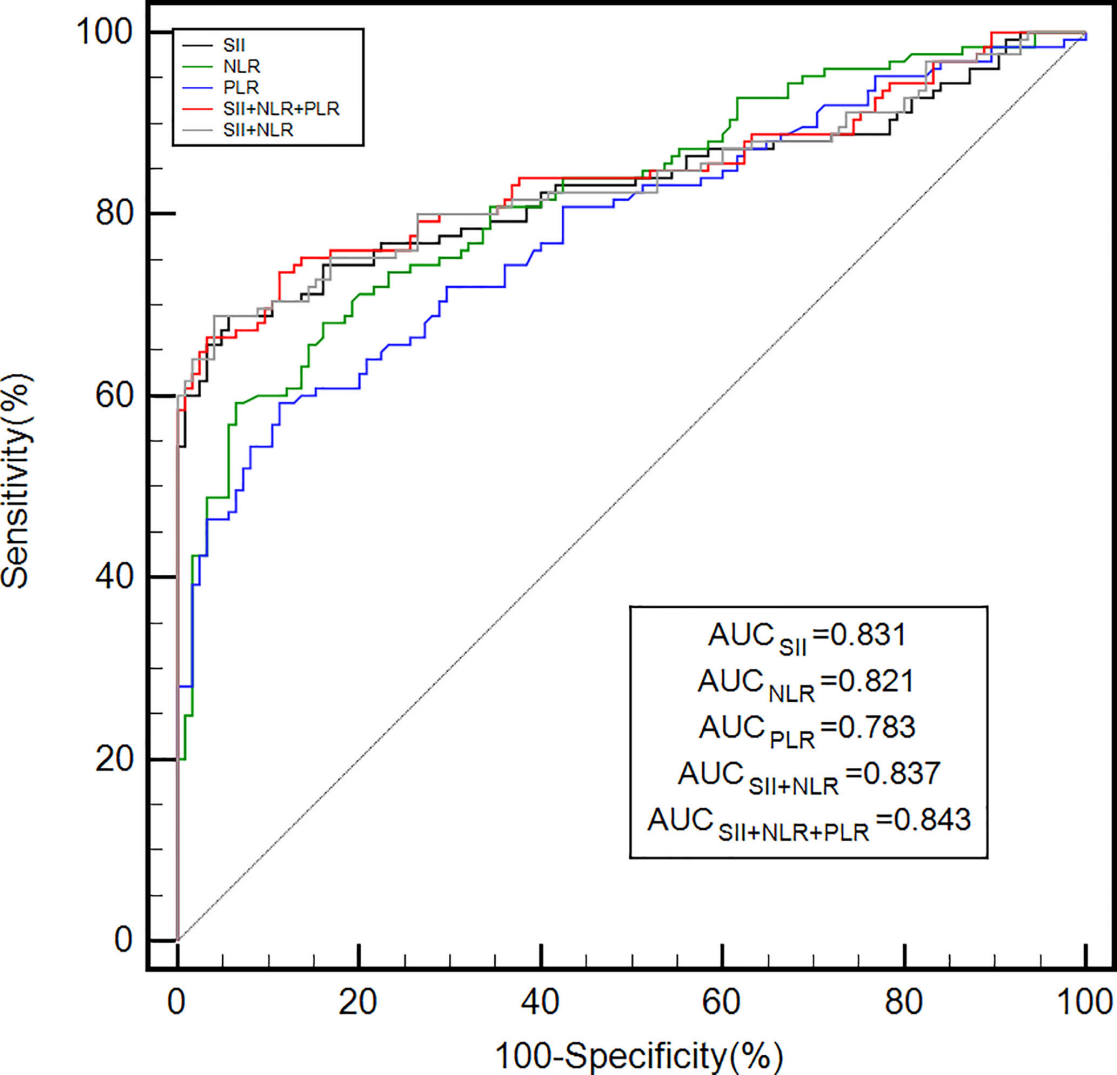
The diagnostic efficiency of the PLR, NLR, and SII alone and in combination for gastric cancer was analyzed by ROC curves. ROC, receiver operating characteristic; AUC, area under the ROC curve; PLR, platelet-lymphocyte ratio; NLR, neutrophil-lymphocyte ratio; SII, systemic immune-inflammation index.

As shown in **Table 2**, the optimal cut-off values for the PLR, NLR, and SII were determined to be 139.5, 2.1, and 438.7, respectively, for GC diagnosis when the Youden index was taken as the maximum value. As shown in **Table 2** and **Figure 1**, the area under the curve (AUC) value of the SII (AUC: 0.831; 95% confidence interval [CI]: 0.779–0.875) was considerably higher than those of the NLR (AUC: 0.821; 95% CI: 0.768–0.866; *P* = 0.016) and PLR (AUC: 0.783; 95% CI: 0.783; *P* = 0.016) when the PLR, NLR, or SII were applied alone to diagnose GC. In addition, the SII had the highest specificity and sensitivity values: 94.40% and 68.80%, respectively. When the three inflammatory indices were combined, the AUC increased to 0.843 (95% CI: 0.791–0.885). The AUC value of the combination of the PLR, NLR, and SII was significantly greater than that of the combination of the SII and NLR (0.837, 95% CI: 0.785–0.880, *P*<0.05), PLR (*P* = 0.020), NLR (*P* = 0.017), or SII alone (*P* < 0.001).

**Table 2:** Diagnostic efficiency of the platelet-lymphocyte ratio, neutrophil-lymphocyte ratio, and systemic immune-inflammation index individually and in combination for gastric cancer patients.

Indicator	AUC (95% CI)	Cut‐off value	Sensitivity (%)	Specificity (%)
SII	0.831 (0.779–0.875)	438.7	68.80	94.40
NLR	0.821 (0.768–0.866)	2.1	59.20	93.60
PLR	0.783 (0.727–0.832)	139.5	59.20	88.80
SII + NLR	0.837 (0.785–0.880)	–	68.80	96.00
SII + NLR + PLR	0.843 (0.791–0.885)	–	66.40	96.80

AUC, area under the receiver operating characteristic curve; CI, confidential interval; PLR, platelet-lymphocyte ratio; NLR, neutrophil-lymphocyte ratio; SII, systemic immune-inflammation index.

### 3.3 Diagnostic value of the PLR, NLR, and SII for early-stage GC

**Figure 2:**
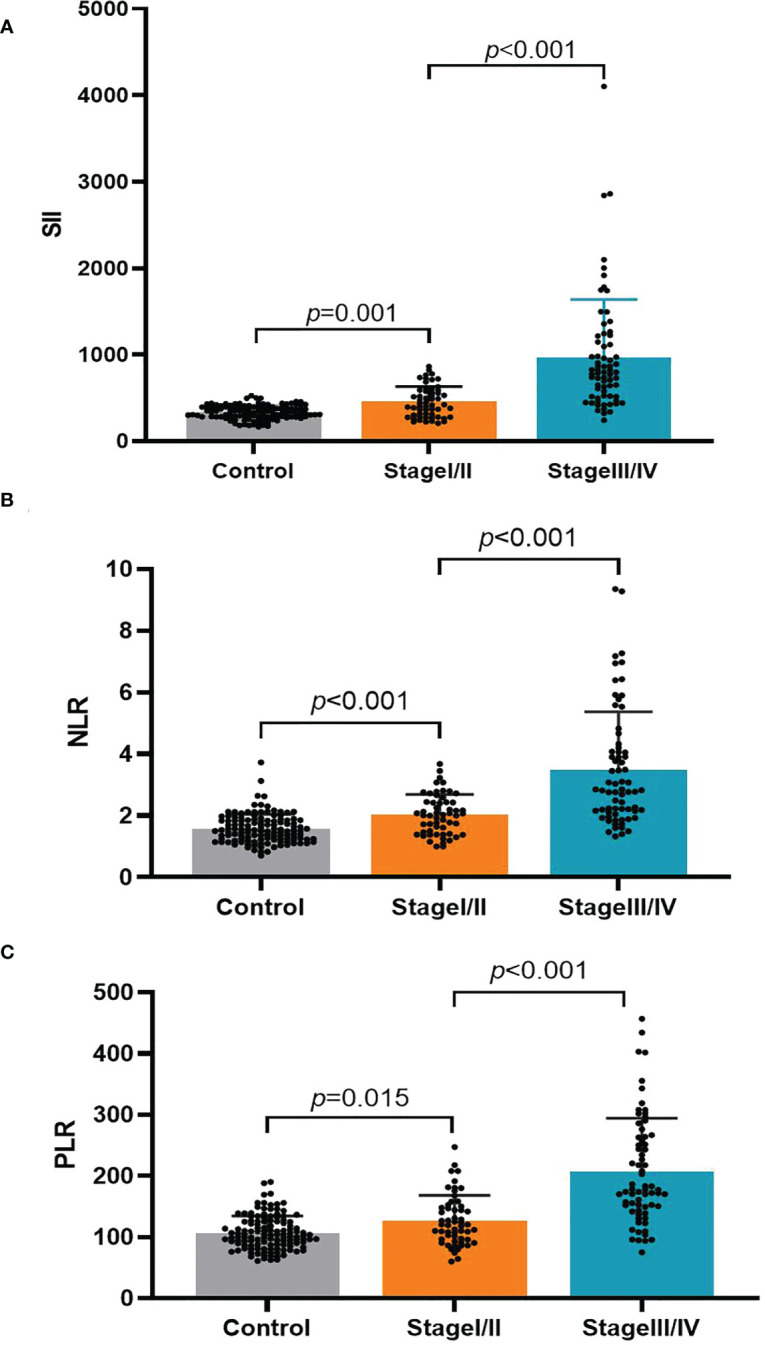
The association of the platelet-lymphocyte ratio (PLR), neutrophil-lymphocyte ratio (NLR), and systemic immune-inflammation index (SII) with the TNM stage in gastric cancer patients. **(A)** SII; **(B)** NLR; **(C)** PLR.

There were three study groups (**Table 3**): the HC group (n = 125, 87 men and 38 women; mean age: 59.86 ± 13.32 years; range: 35–89 years), early-stage (I/II) group (n = 64, 49 men and 15 women; mean age: 61.83 ± 10.34 years; range: 26–81 years), and progressive-stage (III/IV) group (n = 61, 46 men and 15 women; mean age: 62.00 ± 10.31 years; range: 35–82 years). The three groups did not significantly differ in age or sex. We performed an analysis based on TNM staging (**Table 3** and **Figure 2**), which indicated that the PLR, NLR, and SII values were significantly higher in early-stage (I/II) and progressive-stage (III/IV) GC patients than in healthy controls. All three inflammatory indices were notably higher in progressive-stage GC patients than in early-stage GC patients, and the differences were statistically significant (all *P* < 0.05).

**Table 3:** Systemic inflammation laboratory values for various TNM staging.

Variable	Healthy controls	Stage I/II	Stage III/IV
n	125	64	61
Age (years)	59.86 ± 13.32	61.83 ± 10.34	62.00 ± 10.31
Sex (male, %)	87 (69.6%)	49 (76.6%)	46 (75.4%)
SII	339.24 (280.64, 392.33)	417.92 (286.50, 583.08)	796.43 (517.33, 1181.81)
NLR	1.52 (1.23, 1.85)	2.02 (1.42, 2.45)	2.83 (2.14, 4.14)
PLR	103.33 (87.43, 125.50)	119.97 (96.25, 149.56)	178.00 (146.33, 263.38)

PLR, platelet-lymphocyte ratio; NLR, neutrophil-lymphocyte ratio; SII, systemic immune-inflammation index.

### 3.4 Correlations between the PLR, NLR, and SII and GC patients’ clinicopathological characteristics

Relative to the optimal cut-off values, we divided the three inflammatory indices into high- and low-value groups. As displayed in **Table 4**, the PLR, NLR, and SII values were significantly linked with the tumor size, tumor infiltration depth, and lymph node metastasis (all *P* < 0.05). Elevated SII and NLR values were accompanied by distant metastasis (both *P* < 0.001), while the PLR was not linked to distant metastasis (*P* = 0.163). No meaningful correlations existed (*P* > 0.05) between the three indicators and GC patients’ clinicopathological features, such as the degree of tumor differentiation, tumor location, and the presence of lymphatic vessel or nerve infiltration.

**Table 4:** Correlations between gastric cancer patient clinicopathological features and preoperative platelet-lymphocyte ratio, neutrophil-lymphocyte ratio, and systemic immune-inflammation index.

Variable	N	SII		*P*-value	NLR		*P*-value	PLR		*P*-value
		≤ 438.7	> 438.7		≤ 2.1	> 2.1		≤ 139.5	> 139.5	
Tumor size (cm)				< 0.001			< 0.001			0.001
≤ 5	87	37	50		46	41		44	43	
> 5	38	2	36		5	33		7	31	
Tumor differentiation Histologic type				0.843			0.901			0.296
Well/moderate differentiated	40	12	28		16	24		19	21	
Poor/not differentiated	85	27	58		35	50		32	53	
Tumor location				0.245			0.351			0.366
Upper	32	13	19		16	16		15	17	
Middle	51	12	39		21	30		17	34	
Lower	42	14	28		14	28		19	23	
Neural/Lymphovascular invasion				0.726			0.626			0.902
No	58	19	39		25	33		24	34	
Yes	67	20	47		26	41		27	40	
Serosa invasion (T stage)				< 0.001			0.003			< 0.001
T1	28	17	11		18	10		22	6	
T2	18	7	11		10	8		8	10	
T3	25	7	18		10	15		8	17	
T4	54	8	46		13	41		13	41	
Lymph node metastasis (N stage)				< 0.001			< 0.001			< 0.001
N0	42	26	16		28	14		30	12	
N1	16	6	10		10	6		7	9	
N2	25	2	23		3	22		7	18	
N3	42	5	37		10	32		7	35	
Distant metastasis (M stage)				0.001			< 0.001			0.163
M0	106	39	67		51	55		46	60	
M1	19	0	19		0	19		5	14	

PLR, platelet-lymphocyte ratio; NLR, neutrophil-lymphocyte ratio; SII, systemic immune-inflammation index.

## 4 Discussion

Previous studies have indicated that tumor cells can enter the peripheral blood in the early stages of different types of cancer (21). Therefore, circulating tumor cell (CTC) counts in the peripheral blood can possibly be used to diagnose cancer early, understand tumor progression, and assess prognosis (22, 23). However, evaluating CTCs has a limited value in the early diagnosis of various types of cancer because of their rarity and the high cost and complexity associated with the technique (24). A sustained inflammatory response can stimulate tumor growth, invasion, and metastasis (25), and the risk of developing cancer can be reduced with the use of non-steroidal anti-inflammatory drugs (26, 27). Inflammatory immune cells are a crucial component of the tumor microenvironment (7). Reactive oxygen species (ROS) secreted by neutrophils can result in DNA damage, and incomplete or inaccurate repair of genes may lead to carcinogenesis (28). In addition, neutrophils synthesize and secrete oncostatin M and vascular endothelial growth factor (VEGF) to initiate angiogenesis and stimulate tumor growth, thereby furthering the invasion and metastasis of the tumor (29). Lymphocyte activity can reduce tumor cell proliferation and migration rates and plays a crucial part in tumor immunosurveillance (25). The immune response is suppressed following reduced lymphocyte levels, which is directly linked to the occurrence of GC (30). Platelet levels are typically high in cancer patients (31), and activated platelets can promote tumor growth and blood vessel formation through the release of VEGF-integrin (32). Previous studies have shown GC patients have significantly higher neutrophil and platelet counts and significantly lower lymphocyte counts than healthy populations (18, 19). An elevated SII, NLR, or PLR value represents higher levels of inflammation and dysregulation of the immune-inflammation balance in the body, which may be closely related to cancer formation and progression (13, 19). Therefore, the PLR, NLR, and SII may be potential biological indicators for the early diagnosis of GC, as they can indicate the level of inflammation in the body.

In our study, the preoperative SII, NLR, and PLR values were considerably higher in the GC group than in the control group. Compared with the values in the healthy population, the PLR, NLR, and SII were notably elevated in early-stage (stage I/II) GC. This suggests that the preoperative PLR, NLR, and SII could potentially be accurate diagnostic indicators for GC, even at early stages. While evaluating the diagnostic value of the NLR and PLR, Fang et al. (18) learned that these factors were considerably higher in GC patients than in the healthy population, with these differences being more pronounced in the early tumor stages. Similar results were found in colon cancer, where Peng et al. (33) indicated that both the NLR and PLR were considerably higher in early-stage (stage I/II) colon cancer patients than in the healthy population. By plotting ROC curves, we found that applying the SII, NLR, or PLR alone had a better diagnostic value for GC. In addition, the SII had the highest AUC, sensitivity, and specificity values, indicating that the SII individually had the best diagnostic efficacy among the three inflammatory indices examined. This may be because the SII combines three inflammatory cell types, neutrophils, lymphocytes, and platelets, thus allowing for a more integrated and comprehensive representation of the level of inflammation in the body. We subsequently analyzed the diagnostic value of the combination of the PLR, NLR, and SII, which resulted in a higher AUC value than those with all three individually and the combination of SII and NLR. This suggests that the combination of the PLR, NLR, and SII is the most effective for GC diagnosis and may be an important tool that can complement existing diagnostic methods. No previous research has examined the value of the SII in diagnosing GC, nor has any study reported the combined application of the PLR, NLR, and SII for this purpose. Our study verifies the importance of the preoperative SII in GC diagnosis and shows that using the SII, NLR, and PLR in combination has a higher diagnostic value. Our work provides a safer, noninvasive, economical, and simple biological indicator for early GC screening and diagnosis, which can benefit people at high risk of GC.

Furthermore, we found here that the PLR, NLR, and SII differed significantly with the tumor size, invasion depth, lymph node metastasis, and TNM stage, while the SII and NLR were also linked to distant tumor metastasis. These three indices were not correlated with the degree of tumor differentiation and location or the presence/absence of lymphovascular or nerve infiltration. This study reveals the relationship of the SII, NLR, and PLR with GC clinicopathological features, suggesting that inflammation-related indicators may be potential markers of disease progression in GC patients. They may be valuable as a complement to TNM staging for evaluating GC patient survival before surgery and when selecting preoperative treatment options. In a study of 412 GC patients, Hirahara et al. (10) found that patients with higher SII levels had larger tumors, deeper infiltration, increased lymph node metastasis, more advanced TNM stages, and a worse prognosis, consistent with the results of previous studies (10, 11, 15, 16). Similarly, higher NLR and PLR values were associated with more advanced TNM stages in GC patients (19) and shorter postoperative survival time (12, 34, 35). However, there have been few studies on the relationships between the SII and the clinical features of GC, and the conclusions remain controversial. Additional prospective, large-sample, multicenter studies are required to explore this topic in the future.

Most previous research has examined the role of the PLR, NLR, and SII in evaluating the survival of GC patients, while few have explored the value of the PLR and NLR in diagnosing GC. Nevertheless, the value of the SII in diagnosing GC remains unclear. Our study comprehensively analyzed the value of the PLR, NLR, and SII in GC diagnosis. To our knowledge, this is the first study to examine the diagnostic value of combining the PLR, NLR, and SII in GC. However, our study has a few limitations, as it was a single-center study with Chinese participants. As a result, more investigations are required to verify whether the findings can be generalized to other countries and ethnicities. Second, because this study was carried out retrospectively, there may have been a selection bias. Finally, this study only compared patients with GC and healthy individuals. The PLR, NLR, and SII values in individuals with benign gastric disease are unknown, and further research is required to investigate these in the future.

## 5 Conclusions

In summary, the PLR, NLR, and SII have important diagnostic value in GC, including early-stage GC. The SII has the highest efficacy for GC diagnosis when applied individually, while combining all three parameters had the greatest diagnostic value.

## Data availability statement

The raw data supporting the conclusions of this article will be made available by the authors, without undue reservation.

## Ethics statement

The studies involving human participants were reviewed and approved by ethics committee of the Taiyuan Central Hospital of Shanxi Medical University. Written informed consent for participation was not required for this study in accordance with the national legislation and the institutional requirements.

## Author contributions

JZ and ZL were responsible for statistics and data analysis. SD, GL, and HY were responsible for collecting and entering the data. JZ and LZ wrote and revised the manuscript. All authors contributed to the article and approved the submitted version.
