# Corrigendum: Single and combined use of the platelet-lymphocyte ratio, neutrophil-lymphocyte ratio, and systemic immune-inflammation index in gastric cancer diagnosis

**DOI:** 10.3389/fonc.2023.1200951

**Published:** 2023-07-07

**Authors:** Jingliang Zhang, Li Zhang, Shusheng Duan, Zhi Li, Guodong Li, Haiyan Yu

**Affiliations:** ^1^ The First Clinical Medical School, Shanxi Medical University, Taiyuan, China; ^2^ Department of Gastroenterology, Taiyuan Central Hospital of Shanxi Medical University, Taiyuan, China; ^3^ Department of Hematology, Taiyuan Central Hospital of Shanxi Medical University, Taiyuan, China; ^4^ Department of Gastroenterology Surgery, Taiyuan Central Hospital of Shanxi Medical University, Taiyuan, China

**Keywords:** gastric cancer, diagnosis, systemic immune-inflammation index, neutrophil-lymphocyte ratio, platelet-lymphocyte ratio

**Incorrect Affiliation**

In the published article, there was an error in affiliation(s) [Shusheng Duan^3^, Zhi Li^4^]. Instead of “[Shusheng Duan^3^, Zhi Li^4^]”, it should be “[Shusheng Duan^1^, Zhi Li^3^]”.

The authors apologize for this error and state that this does not change the scientific conclusions of the article in any way. The original article has been updated.

